# Thin-Film Composite Polyamide Membranes Modified with HKUST-1 for Water Treatment: Characterization and Nanofiltration Performance

**DOI:** 10.3390/polym17091137

**Published:** 2025-04-22

**Authors:** Roman Dubovenko, Mariia Dmitrenko, Anna Mikulan, Margarita Puzikova, Ilnur Dzhakashov, Nadezhda Rakovskaya, Anna Kuzminova, Olga Mikhailovskaya, Rongxin Su, Anastasia Penkova

**Affiliations:** 1 St. Petersburg State University, 7/9 Universitetskaya nab., St. Petersburg 199034, Russia; st097675@student.spbu.ru (A.M.); st095099@student.spbu.ru (M.P.); st117543@student.spbu.ru (I.D.); st094879@student.spbu.ru (N.R.); a.kuzminova@spbu.ru (A.K.); st113220@student.spbu.ru (O.M.); 2 State Key Laboratory of Chemical Engineering, School of Chemical Engineering and Technology, Tianjin University, Tianjin 300072, China; surx@tju.edu.cn

**Keywords:** thin-film composite, metal–organic framework, interfacial polymerization, membrane, nanofiltration, dye

## Abstract

The development of sustainable nanofiltration membranes requires alternatives to petroleum-derived polymer substrates. This study demonstrates the successful use of an eco-friendly cellulose acetate/cellulose nitrate (CA/CN) blend substrate for fabricating high-performance modified thin-film composite (mTFC) membranes. A dense, non-porous polyamide (PA) selective layer was formed via the interfacial polymerization method and modified with 0.05–0.1 wt.% HKUST-1 (Cu_3_BTC_2_*,* MOF-199). Characterization by FTIR, XPS, SEM, AFM, and contact angle measurements confirmed the CA/CN substrate’s suitability for TFC membrane fabrication. HKUST-1 incorporation created a distinctive ridge-and-valley morphology while significantly altering PA layer hydrophilicity and roughness. The mTFC membrane performance could be fine-tuned by the controlled incorporation of HKUST-1; incorporation through the aqueous phase slowed down the formation of the PA layer and significantly reduced its thickness, while the addition through the organic phase resulted in the formation of a denser layer due to HKUST-1 agglomeration. Thus, either enhanced permeability (123 LMH bar^−1^ with 0.05 wt.% aqueous-phase incorporation) or rejection (>89% dye removal with 0.05 wt.% organic-phase incorporation) were achieved. Both mTFC membranes also exhibited improved heavy metal ion rejection (>91.7%), confirming their industrial potential. Higher HKUST-1 loading (0.1 wt.%) caused MOF agglomeration, reducing performance. This approach establishes a sustainable fabrication route for tunable TFC membranes targeting specific separation tasks.

## 1. Introduction

The problem of environmental pollution is worsening every year, as the amount of industrial emissions and household waste continues to increase with rapid industrialization and urbanization. This issue particularly concerns the discharge of organic and inorganic pollutants into wastewater, which adversely affects the planet’s natural ecosystems [1]. Some of the main contaminants are dyes, which are actively used in the textile, paper and pulp, leather, paint, cosmetic, and food industries [2,3]. For instance, Congo Red, a diazo dye, poses severe risks when released into water sources, as it can infiltrate the food chain and potentially lead to carcinogenic effects [4]. Similarly, Sun Yellow has raised significant concerns regarding its environmental and health impacts. Associated health risks include potential allergic reactions, hyperactivity in children, and possible carcinogenic effects at high doses [5], prompting regulatory agencies worldwide to impose usage limits [6]. Additionally, M. Olgun et al. [7] reported that, despite the known harmful effects of Alphazurine (Brilliant blue), the existing controls and regulations concerning the legally permitted quantities are insufficient. The majority of industry dyes are toxic and chemically stable, persisting in the environment due to their resistance to biological degradation. For this reason, it is crucial to develop effective wastewater treatment technologies for dye-contaminated water.

There are various methods for removing dyes from water, including adsorption [8], membrane separation [9], coagulation [10], flocculation [11], and oxidative methods [12]. Among these, membrane separation methods demonstrate exceptional potential due to their ease of operation, low energy consumption, ability to function at room temperature, absence of phase transition, and provision of a natural cleaning process without the use of chemicals [13,14]. Recently, nanofiltration (NF), which occupies an intermediate position between ultrafiltration (UF) and reverse osmosis, has garnered significant research interest due to its considerable efficiency, as evidenced by its widespread application in water and alcoholic solution purification from dyes, heavy metals, bacteria, viruses, microorganisms, colloidal particles, and organic compounds and its use in various industries [15]. The rapid advancement of NF technology creates a demand for the development of new efficient membrane materials that possess enhanced transport properties and/or environmental safety.

Membranes are commonly classified as polymeric and ceramic according to the materials used in their preparation [16]. While ceramic membranes are characterized by their tunable pore structure and exceptional stability, allowing them to be used for complex water purification processes, the large-scale application of ceramic membranes is limited due to their high capital costs [17]. Polymeric membranes are currently the most widely used, largely due to their high technological efficiency and low cost [18]. Based on the separation mechanisms, NF membranes can be categorized into the following five different types: positively charged, negatively charged, mixed-charge NF membranes, sieving-type NF membranes, and biomimetic NF membranes. Among these, positively and negatively charged NF membranes can be further divided based on their interfacial structure, known as integral asymmetric NF membranes and thin-film composite (TFC) membranes [19].

Among the various materials used for manufacturing membranes, TFC membranes dominate industrial applications for NF and reverse osmosis due to their superior rejection capabilities and high water flux [20,21]. These membranes, as a rule, are fabricated by depositing a selective polyamide (PA) layer onto a mechanically supportive porous substrate through the interfacial polymerization (IP) method, wherein two monomers react at the interface of two immiscible phases on the substrate’s surface [22]. However, membrane performance typically exhibits an inverse relationship between permeability and selectivity, where higher permeability generally corresponds to lower rejection [23,24]. This trade-off negatively impacts industrial application efficiency by increasing both energy consumption and operational costs. Recent advances have led to the development of novel materials demonstrating both exceptional permeability and selectivity, challenging traditional performance trade-offs [25]. In particular, modified TFC (mTFC) membranes incorporating various fillers within the PA layer represent a promising approach to overcome this limitation. Various modifiers employed in the production of mTFC membranes have been described in the literature, including metal oxides [26,27], carbon quantum dots [28,29], silicon dioxide [30], carbon nanotubes [31], inorganic salts [32], and graphene oxide [33], all aimed at enhancing separation efficiency. Additionally, metal-organic frameworks (MOFs) have also garnered significant attention. MOFs—including representative structures developed at leading research institutions such as UiO (University of Oslo), MIL (Materials of the Institut Lavoisier), and HKUST (Hong Kong University of Science and Technology)—have shown great promise as performance-enhancing modifiers for membrane applications. These crystalline porous materials offer the following two key advantages for membrane modification: (1) exceptionally high specific surface areas, and (2) precisely tunable porosity that can be systematically controlled through rational ligand selection and framework [34,35,36,37]. For instance, in the study [38], the introduction of a Zn-based MOF into a polyphenylsulfone support led to an mTFC membrane that demonstrated remarkable retention (90% for NaCl) and productivity (2.46 LMH bar^−1^) during desalination. Similarly, an mTFC membrane modified with Cr-based MIL-101 was utilized for the nanofiltration of methanolic dye solutions, which resulted in over a twofold increase in permeability (up to 12.03 LMH bar^−1^) while maintaining retention for Rose Bengal dye (98.15%, 1017 g mol^−1^) [39]. An mTFC membrane with Zr-based UiO-66-SO_3_H was successfully employed to separate toxic ions like Cd and Hg (9.57 LMH bar^−1^, up to 90%) [40]. Among the various MOFs, HKUST-1 is particularly noteworthy as an effective modifier for the PA layer in mTFC membranes, as demonstrated in studies by other researchers [41,42,43,44]. Its unique porous structure, stable chemical properties, and abundant open ligand sites facilitate favorable interactions with the polymer matrix and PA layer [45,46]. Importantly, HKUST-1 is commercially available, offering significant potential for the practical application of academic research in industrial settings. Among the various MOFs, in this study, HKUST-1 was chosen as a modifier for the development of TFC membranes due to its porous structure, suitable pore size (0.5–1.4 nm), hydrophilicity, commercial availability, and strong affinity for the PA layer [42,47,48,49,50]. This selection is further supported by recent works, which have demonstrated that HKUST-1 incorporation promotes additional intramolecular hydrogen bonding within the polyamide matrix, thereby enhancing membrane performance [51], and it enables the formation of strong hydrogen-bonding interactions between the HKUST-1 ligand and polymer hydroxyl groups of substrate [52].

A recent review by M. Kahrizi [53] highlighted the importance of selecting an appropriate modifier and substrate with small surface pore sizes, narrow surface pore size distributions, and optimal hydrophilicity (with water contact angle values appearing to range between 40° and 60°) to create an effective mTFC membrane.

Thus, in this study, we selected a porous membrane based on a blend of hydrophilic cellulose derivatives (cellulose nitrate (CN) and cellulose acetate (CA)) as an optimal substrate for manufacturing modified thin-film composite (mTFC) membranes. This approach successfully combines environmental sustainability with excellent functional properties [45,46,47], addressing growing concerns in membrane technology regarding the use of fossil-based, toxic solvents, monomers, polymers, cross-linkers, and other conventional additives [54]. The membrane industry faces increasing pressure to replace these environmentally harmful materials with renewable and sustainable alternatives. Bio-sourced materials like cellulose derivatives offer particularly attractive solutions due to their high biodegradability, which can significantly reduce plastic waste pollution [55]. Furthermore, to the best of our knowledge, there are no existing reports in the literature on the use of CA/CN blend membranes (substrates) for the fabrication of TFC membranes. CN is recognized for its strong affinity for proteins, especially for amide linkages [56], which facilitates good adhesion between the PA layer and the CN-based substrate. Additionally, to enhance the mechanical properties, CA was utilized in a blend with CN to produce a substrate with tailored properties and long-term effectiveness [57].

Thus, this study addresses the research gap in developing efficient nanofiltration TFC membranes for enhanced dye wastewater treatment, utilizing cellulose-derived substrates, including cellulose nitrate, which has an affinity for amide fragments. TFC membranes were created using the IP technique to form a selective PA layer modified with synthesized HKUST-1 onto a blend of CN and CA substrate, emphasizing ecological sustainability. Additionally, the investigation focused on the impact of MOFs on the characteristics of mTFC membranes, exploring the relationships among structure, morphology, and membrane properties to provide insights into performance mechanisms. The influence of MOFs was studied through (i) their incorporation into aqueous and/or organic phases during PA layer formation and (ii) variations in MOF concentration. The synthesized HKUST-1 and the resulting membranes were characterized using techniques such as Fourier-transform infrared spectroscopy, X-ray diffraction, X-ray photoelectron spectroscopy, low-temperature nitrogen adsorption, scanning electron and atomic force microscopies, and contact angle measurements.

## 2. Materials and Methods

### 2.1. Materials

A blend of cellulose diacetate (CA, Mn = 40,000 g mol^−1^, CJSC “Vladipor”, Vladimir, Russia) and cellulose nitrate (CN, Mn = 43,000 g mol^−1^, nitrogen content 11.91%, “Hengshui Heshuo Cellulose Co., Ltd.”, Hengshui, China) was selected as a matrix for the porous membrane (substrate) preparation. A non-woven polyester support (Novatexx2430) from Freudenberg Filtration Technologies (Weinheim, Germany) was used as the backing material for the preparation of the porous CA/CN substrate. N,N-Dimethylacetamide (DMAc, puriss.) from Vecton (Saint Petersburg, Russia) was used as the solvent without further purification. An aqueous solution of piperazine (PIP, Piperazine Anhydrous, 98%, pure, Sisco Research Laboratories Pvt., Ltd., Taloja, India) and a solution of trimellitic anhydride chloride (TMC, 1,3,5-benzenetricarbonyl trichloride, 98%, pure, Tokyo Chemical Industry Co., Ltd., Tokyo, Japan) in naphtha (Naphtha C4-155/200, LLC “Vershina”, Vsevolozhsk, Russia) were used for IP to form a PA layer on a porous substrate.

A copper-based metal-organic framework (MOF) (HKUST-1 with a specific BET surface area of 171.6 ± 0.9 m^2^ g^−1^ and benzene-1,3,5-tricarboxylic acid as a ligand) was applied as a PA layer modifier. The synthesis procedure and characterization of HKUST-1 are presented in the Supplementary Materials (Sections S1–S3, Figures S1–S4).

To investigate the transport characteristics of the thin film membranes, aqueous solutions of the following dyes were employed: Sunset Yellow (SY, E110), Congo Red (CR, E129), and Alphazurine (AZ, E133). The structures, absorption maxima, and molar mass of the dyes are presented in Table 1. The molecule sizes were also assessed using Van der Waals radii and analyzed with Chemcraft (Figure S5 of the Supplementary Materials).

### 2.2. Thin Film Membrane Preparation

#### 2.2.1. Porous Substrate Preparation

The calculated amounts of CN and CA were dissolved in DMAc stirring for 4 h, followed by ultrasonic treatment to obtain a 20 wt.% CA/CN (1:3) solution. Flat sheet porous CA/CN (1:3) substrate was produced by the phase inversion method with a non-solvent induced phase separation (NIPS) technique [58,59]. The polymer solution was applied through a casting blade with a gap width of 200 µm onto a non-woven polyester support, which was fixed to a glass plate, which was then immersed in a coagulation bath containing distilled water at 25 °C. After 24 h, the porous support was ready for further surface modification via IP.

#### 2.2.2. Formation of PA Layer on a Porous Substrate

To stabilize the prepared CA/CN-20 (1:3) porous substrate (S), it was pre-treated by soaking in distilled water at 50 °C for 20 min, followed by transferring to a bath with distilled water for 30 min at room temperature. The resulting substrate was secured onto a glass plate. The PA layer was formed by sequentially applying a 4 wt.% aqueous solution of PIP and a 0.24 wt.% solution of TMC in naphtha onto the substrate. After 5 s, excess of each solution was removed using a rubber squeegee. The resulting thin film membrane with the deposited PA layer was placed in an oven at 50 °C for 10 min. Then, it was transferred to a bath with distilled water at 50 °C until fully cooled.

Modification of the thin film membrane with MOF particles was carried out depending on the method of introducing the modifier into the PA matrix. The calculated amount of HKUST-1 was added to the 4 wt.% aqueous solution of PIP (water phase, W) and/or the 0.24 wt.% solution of TMC in naphtha (organic phase, O) by stirring for 2 h. The resulting composite solutions were subjected to ultrasonic treatment for 60 min and then were used to form the PA layer according to the previously described method. The fabricated membranes were designated, corresponding to their respective compositions (Table 2).

### 2.3. Membrane Performance in Nanofiltration

**Figure 1:**
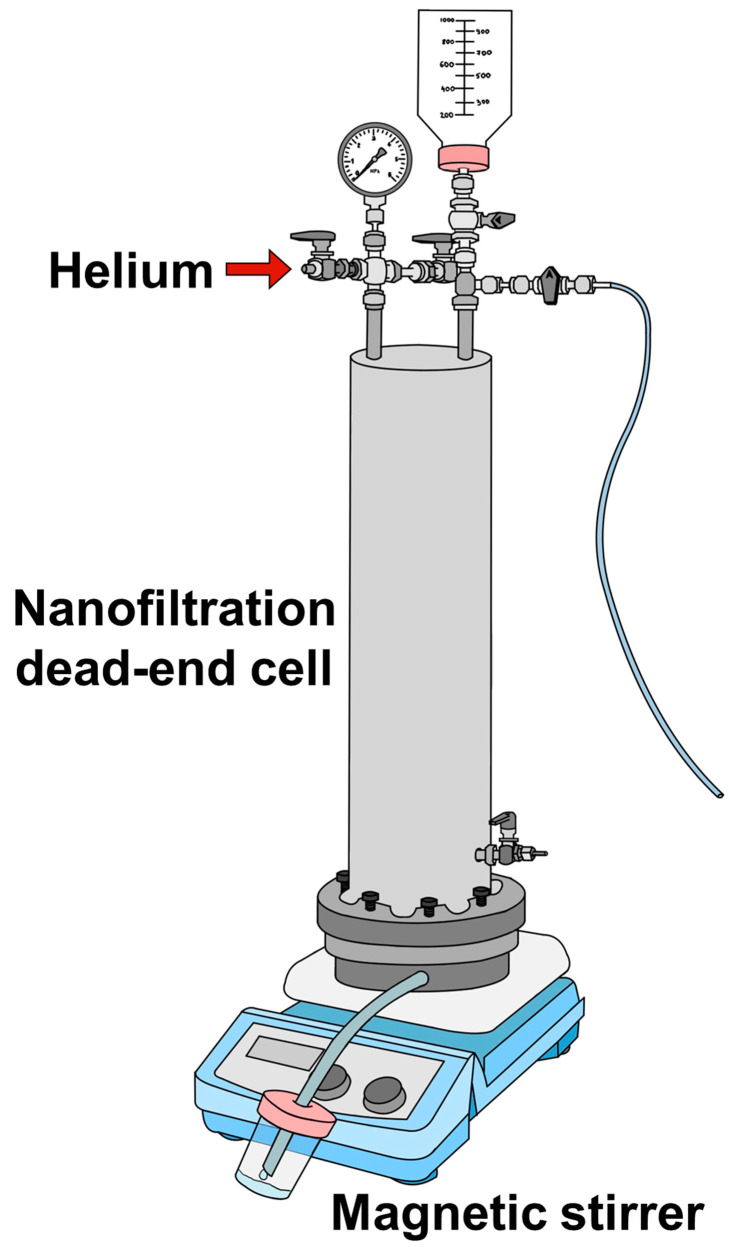
The nanofiltration dead-end setup.

The transport characteristics of the substrate and thin film membranes were investigated by nanofiltration for the separation of dyes. A dead-end laboratory cell (NPK BIOTEST, Kirishi, Russia) with an effective membrane area of 0.2 × 10^−2^ m^2^ was utilized at room temperature and a transmembrane pressure of 1 bar. A schematic diagram of the setup with the nanofiltration cell is presented in Figure 1.

Nanofiltration was conducted using aqueous solutions of dyes at a concentration of 0.01 g L^−1^, including SY, CR, and AZ. To prevent concentration polarization, the solutions were continuously stirred. The analysis of dye concentrations in both the feed and permeate was performed using a PE-5400UV spectrophotometer (EKROSHIM, St. Petersburg, Russia) at wavelengths, corresponding to the absorption maxima for each dye (Table 1). Additionally, the effectiveness of nanofiltration for solutions containing heavy metal ions was evaluated to assess potential industrial applications. The test solutions consisted of Cu(NO_3_)_2_, Pb(NO_3_)_3_, and Cd(NO_3_)_3_ at a concentration of 50 mg L^−1^ [60]. Between measurements, the filtration cell was rinsed with a 5 g L^−1^ solution of Trilon B. The concentrations of heavy metal ions in the feed and permeate solutions were precisely determined using stripping voltammetry (TA-4 voltammetric analyzer, Tomanalit, Tomsk, Russia). The electrochemical setup comprised silver chloride electrodes as the reference and auxiliary electrodes, with a mercury film electrode serving as the working electrode.

The membrane permeability (*L*, L m^−2^h^−1^bar^−1^, LMH bar^−1^), as the ratio of permeation flux (*J*) to the transmembrane pressure (∆*P*, bar) (Equation (1)), and rejection coefficients (*R*, %) (Equation (2)) were calculated as follows [61]:

(1) $$L=\frac{J}{\Delta P}=\frac{V}{A\;t\;\Delta P},$$

where *V* is the permeate volume (L), *t* is the permeate collection time (h), and *A* is the effective surface area of the membrane (m^2^).

(2) $$R=\left(1-\frac{C_{p}}{C_{f}}\right)\times 100\%,$$

where *C_p_* and *C_f_* are the content of the component in the permeate and feed, respectively.

Nanofiltration experiments were carried out for at least a week for each membrane. The transport characteristics were assessed through a series of measurements conducted in triplicate to ensure the accuracy and reproducibility of the results. Each parameter was averaged by calculating the mean value. To quantify the precision of measurements, the standard deviation and relative standard deviation were calculated for each set of data. The overall measurement error and confidence intervals were determined using a normalized normal distribution.

### 2.4. Fourier-Transform Infrared Spectroscopy

The structure of the developed substrate and thin film membranes with the PA and PA/MOF layer was studied by Fourier-transform infrared spectroscopy (FTIR) using an IRAffinity-1S spectrometer (Shimadzu, Kyoto, Japan) and an attenuated total reflection (ATR) accessory (PIKE Technologies, Moscow, Russia) in the range of 450–4000 cm^−1^ at 25 °C.

### 2.5. Scanning Electron Microscopy

The morphology of the substrate and membranes, including the surface and cross-section, was studied using a scanning electron microscope Auriga Laser (Carl Zeiss GT, Oberkochen, Germany). Membrane cross-section samples were coated with carbon using a vacuum sputter coater.

### 2.6. Atomic Force Microscopy

The surface topographical characteristics of the substrate and membrane samples were analyzed by atomic force microscopy (AFM). These measurements were carried out using an NT-MDT Ntegra Maximus atomic force microscope (NT-MDT Spectrum Instruments, Moscow, Russia) in tapping mode. Standard silicon cantilevers, featuring a spring constant of 15 N·m^−1^, were utilized for the analysis. Measurements were performed at least five times over an area of 10 × 10 μm.

### 2.7. X-Ray Photoelectron Spectroscopy

X-ray photoelectron spectroscopy (XPS) analyses were conducted using an “Escalab 250Xi” photoelectron spectrometer (Thermo Fisher Scientific Inc., Waltham, MA, USA), which operated with Al Kα radiation (photon energy of 1486.6 eV). The spectra were acquired in constant pass energy mode, with a pass energy of 100 eV for survey spectra and 50 eV for elemental core level spectra, utilizing an XPS spot size of 650 μm. The overall energy resolution for the measurements was approximately 0.3 eV. The studies were carried out at room temperature in an ultrahigh vacuum environment, around 1 × 10^−9^ mbar. An ion-electronic charge compensation system was used to neutralize the sample charge during the XPS measurements.

### 2.8. Measurement of Contact Angle

To analyze the hydrophilic–hydrophobic balance of the surfaces of substrate and TFC membranes, contact angles were determined by the attached bubble method and the sessile drop techniques, respectively. Measurements were conducted using an LK-1 goniometer (Open Science Research and Production Company, Krasnogorsk, Russia). Data analysis was carried out using the Drop Shape software (version 1, Laboratory of Mathematical Methods of Image Processing, Lomonosov Moscow State University, Moscow, Russia).

## 3. Results and Discussions

### 3.1. TFC Membrane Performance

**Figure 2:**
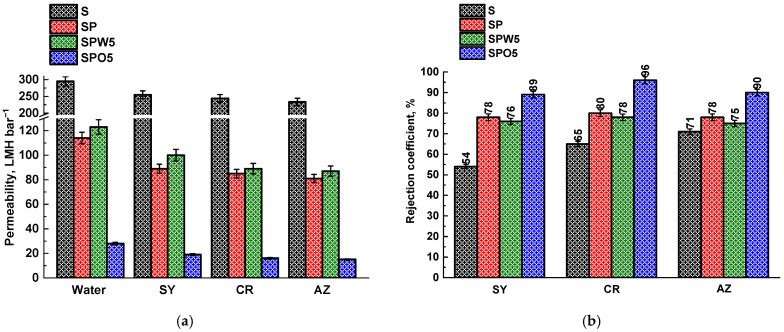
The permeability of water and dye solutions (**a**) and rejection coefficients (**b**) for the unmodified S substrate and TFC membranes.

The IP method was employed to create the NF TFC membranes (SP, SPO5, and SPW5). To study the influence of MOF as a modifier, the effect of introducing MOF into different phases was investigated; namely, HKUST-1 was added to an aqueous solution of PIP (the SPW5 membrane) and/or an organic solution of TMC (the SPWO5+5 and SPO5 membranes) to form a composite selective PA layer onto the S substrate, with different concentrations of the modifier on the transport characteristics of membranes in the nanofiltration of dye solutions. The performance of the unmodified S substrate and TFC membranes with a thin PA and PA/MOF layer (SP, SPO5 and SPW5) is presented in Figure 2.

It was demonstrated that there was a significant increase in rejection coefficients and a decrease in permeability for the SP membrane compared to the S substrate, confirming the successful formation of a PA layer (also confirmed by FTIR, SEM, and XPS data). The change in rejection coefficients was attributed to the negative charge of the PA membrane surface, which allowed it to exclude anionic dyes through electrostatic repulsion between the selective layer and the dyes [62]. The introduction of HKUST-1 into the aqueous phase (the SPW5 membrane) resulted in an increase in permeability and a minor reduction in dye rejection (no more than 3%). The improved permeability of the SPW5 membrane could be attributed to the introduction of porous HKUST-1 (with pore diameters up to 1.4 nm [49]), which supplied additional transport channels for water passing through the membrane [63] and caused the enhanced hydrophilicity, the roughness of the SPW5 membrane surface (confirmed by AFM and contact angle data), and the decreased thickness of the forming PA layer (confirmed by SEM). On the other hand, this reduction in the PA layer thickness, combined with the porous structure of the MOF, resulted in a slight decrease in the retention capacity of the SPW5 membrane due to the penetration of dye molecules through the membrane (the sizes of the dye molecules are presented in the Supplementary Information, Figure S5) [23]. The utilization of the SPO5 membrane resulted in a four-fold decrease in permeability compared to the SP membrane, accompanied by more than an 11% increase in rejection coefficients. The decrease in the SPO5 permeability was caused by the MOF agglomeration [64] and surface defects, such as voids and pinholes, (confirmed by SEM) due to the dissolution of HKUST-1 in a non-polar solvent (naphtha) [65,66], resulting in an increase in the overall hydraulic resistance of the membrane. Additionally, the observed decreased permeability values may indicate an intrusion of PA into the substrate, as suggested in the works [67,68].

**Figure 3:**
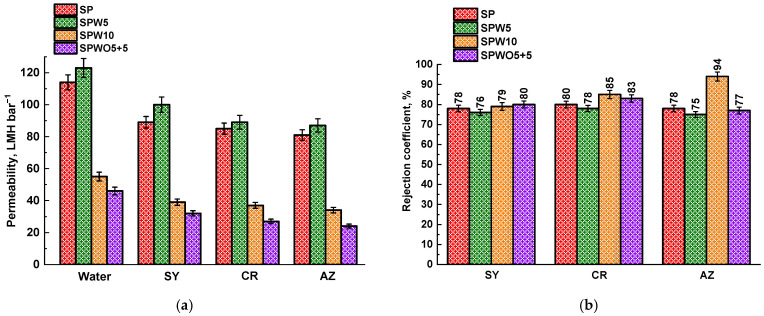
The permeability of water and dyes (**a**) and rejection coefficients (**b**) for unmodified and modified TFC membranes.

To determine the optimal composition of the PA layer and vary the conditions to study the effect of different MOF introduction approaches, the SPW10 (with 1 wt.% HKUST-1 introduced into water phase) and SPWO5+5 (with 0.05 wt.% HKUST-1 introduced into both aqueous and organic phases during the formation of the PA layer) membranes were also studied. Figure 3 shows the permeability and rejection coefficients for the SPW10 and SPWO5+5 mTFC membranes, as well as those of the SP and SPW5 membranes for comparison.

When the MOF concentration reached 0.1 wt.%, the water permeability decreased to 55 and 46 LMH bar^−1^ for the SPW10 and SPWO5+5 membranes, respectively. The utilization of an excess amount of HKUST-1 during the IP process induced significant morphological alterations, including increased PA layer thickness and the formation of a “ridge and valley-nodular” structure (as illustrated by SEM). These modifications enhanced mass transfer resistance while also contributing to the observed permeability decline, which could be also attributed to both non-uniform MOF particle distribution across the SWP10 PA layer and partial obstruction of transport channels, consistent with the previous report [69]. Surface characterization revealed distinct morphological differences between SPWO5+5 and SPO5 membranes. While SPWO5+5 exhibited surface topography similar to SPO5, its PA-based layer had nodular protrusions and an increased thickness of 740 nm (confirmed by SEM). This suggests reduced PA intrusion into the substrate, explaining both the enhanced permeability and compromised rejection coefficients compared to SPO5 (Figure 2). In contrast, the SPW10 membrane demonstrated superior rejection capabilities relative to SPW5, coupled with its well-developed surface morphology and highest average roughness values (excluding SPWO5+5), as verified by AFM. The pronounced surface roughness appeared to significantly enhance the size-sieving effect, evidenced by progressively improved dye rejection correlating with molecular weight increase.

Thus, based on the obtained data, the optimal TFC membranes for the nanofiltration of dye solutions were determined to be the SPW5 membrane, characterized by enhanced permeability, and the SPO5 membrane, which possessed high rejection coefficients. Depending on the separation tasks, it is possible to use one or the other membrane by only varying the method of introducing the MOF during the preparation process, namely to increase the productivity or selectivity of the process of water purification of dyes.

The relationship between permeability and pressure was determined to evaluate membrane stability at different pressures ranging from 1 to 10 bar (Figure S6 of Supplementary Materials). The results demonstrated that an increase in pressure led to an almost linear rise in permeation flux for all membranes, confirming their suitability at a high pressure. The observed deviation from a strictly linear trend may be attributed to pore deformation during the compaction of the CA/CN-20 (1:3) porous substrate over time [70]. Notably, the most significant deviations from linearity occurred at pressures exceeding 6 bar, which may represent the optimal pressure range for process intensification. This pressure threshold appears to balance flux enhancement with membrane structural stability.

**Figure 4:**
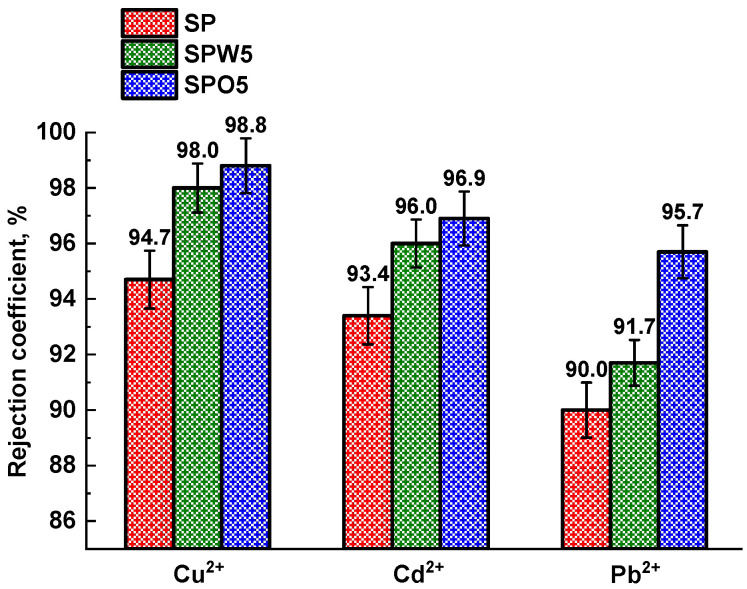
The rejection coefficients of heavy metal ions for unmodified and modified TFC membranes.

The SP, SPW5, and SPO5 membranes were further evaluated for nanofiltration performance using aqueous solutions containing heavy metal ions to assess their potential for industrial wastewater treatment applications (Figure 4). Throughout these experiments, the membranes maintained permeability values consistent with the water permeability data shown in Figure 2.

The SPO5 membrane exhibited the highest selectivity (98.8% for Cu^2+^, 96.9% for Cd^2+^, and 95.7% for Pb^2+^). The separation efficiency of heavy metals was found to be influenced by both Donnan exclusion and size-sieving effects. Specifically, the hydration diameters of the ions follow this descending order: Cd^2+^ > Cu^2+^ > Pb^2+^ [71,72]. Nevertheless, the highest separation efficiency was observed for Cu^2^⁺, which can be attributed to the additional contribution of membrane surface charge properties, influenced by the pH of the feed solution. Based on base dissociation constants (pKb), the acidity of the metal salt solutions increased in the following order: Pb^2+^ > Cu^2+^ ≫ Cd^2+^. Notably, the aqueous Cu(NO_3_)_2_ solution was more acidic than those of Cd^2+^, resulting in a higher zeta potential of the membrane surface [72]. The synergistic effect of these two factors (Donnan exclusion and hydration radius) contributed to the superior separation of Cu^2+^.

The incorporation of MOFs into the PA layer enhanced the rejection capability of the mTFC membranes, likely due to the HKUST-1 adsorption of heavy metal ions, as reported in previous studies [73,74]. Consequently, the SPO5 membrane demonstrated the highest rejection coefficients (>95.7%) for all tested metal ions, consistent with the mechanisms discussed above.

### 3.2. Structure and Physicochemical Properties

**Figure 5:**
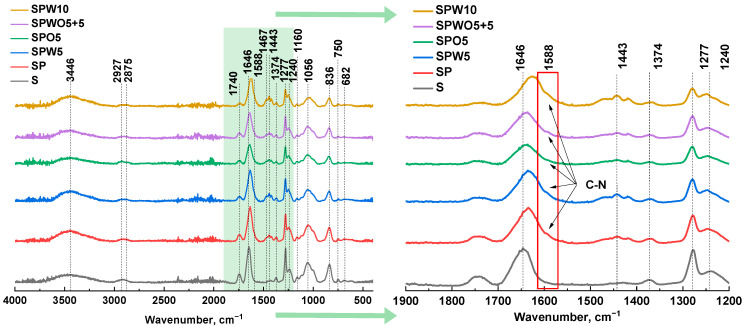
FTIR spectra of the S and TFC (SP, SPO, SPW, and SPWO) membranes.

Structural characteristics and physicochemical properties were investigated for the substrate (S) and TFC membranes (SP, SPO, SPW, and SPWO) by various analysis methods. Figure 5 presents the FTIR spectra.

Based on the FTIR spectra, characteristic peaks of CA were identified, reflecting the stretching vibrations of the ester group C=O and C-H from CH_3_ at 1740 cm^−1^ and 2927 cm^−1^, respectively. The peaks at 1374 cm^−1^ and 1240 cm^−1^ represent the deformation vibrations of the -CH_3_ group, while the peak at 1056 cm^−1^ is attributed to ether functional groups C-O-C [75]. For CN, the peaks at 1646 cm^−1^ and 1277 cm^−1^ indicate the asymmetric and symmetric stretching vibrations of the nitro groups NO_2_, whereas the peaks at 836 cm^−1^, 750 cm^−1^, and 682 cm^−1^ correspond to the stretching vibrations ν(N-O), bending vibrations γ_w_(NO_2_), and scissoring vibrations δ(NO_2_), respectively [76]. Additionally, the peaks at 3446 cm^−1^ and 1467 cm^−1^ reflect stretching and deformation vibrations of the O-H group. The peak at 2875 cm^−1^ is associated with the stretching vibrations of C-H (CH_2_) for both CA and CN [75,77].

FTIR spectroscopy confirmed the successful formation of a PA layer on the surface of the porous substrate. For TFC membranes in the spectra, characteristic maxima in the region of 1443 cm^−1^ were observed, corresponding to the vibrations of C=C from the aromatic ring of the TMC fragment [78], and 1588 cm^−1^ corresponds to the amide group C–N in-plane bending [75]. The shift of the vibration wavelength from 1646 cm^−1^ to the range of 1635-1623 cm^−1^ indicates the involvement of the nitro group in binding with the amide group. Comparing the membrane spectra with that of HKUST-1 (Figure S4 of Supplementary Materials), no peaks corresponding to the vibrations of the MOF were observed, which could be due to its low concentration.

**Figure 6:**
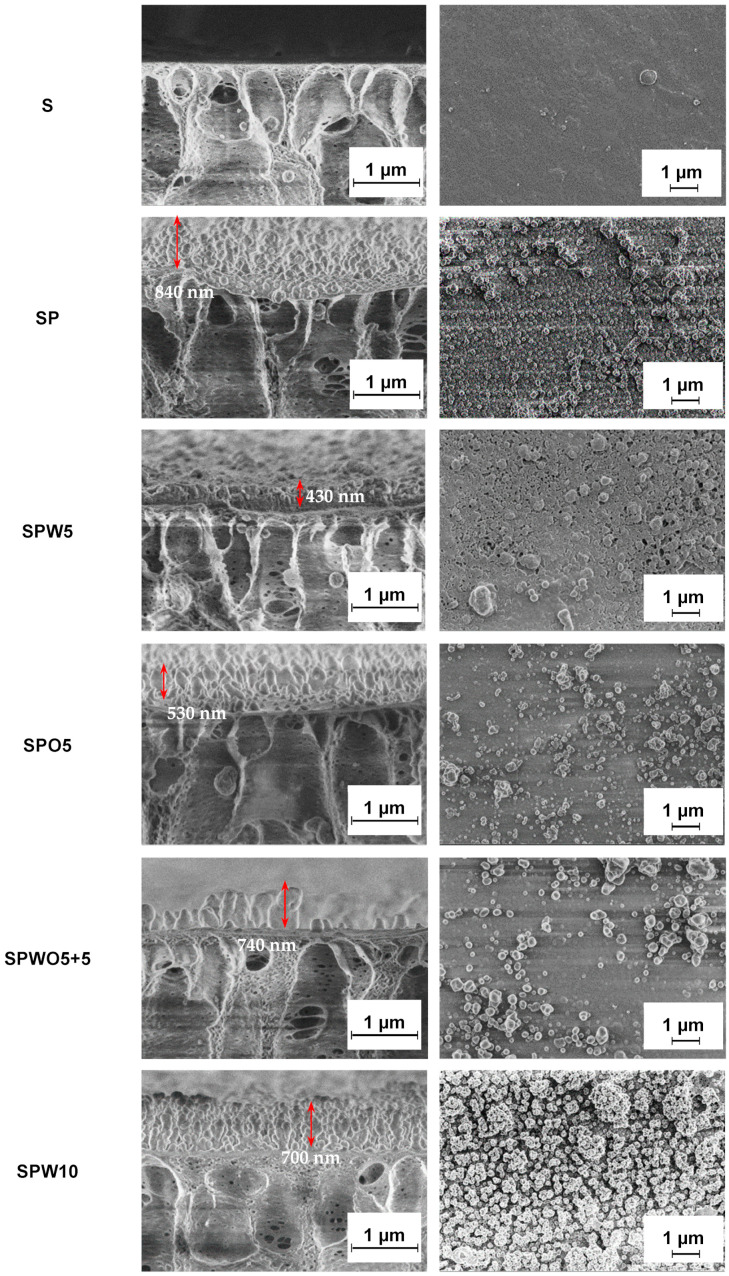
The SEM micrographs of the cross-section and the surface of the S substrate and TFC membranes.

The morphology of the S substrate and TFC membranes was investigated by SEM. Figure 6 presents microphotographs of the cross-section and surface of the membranes.

The cross-section structure of the S substrate is characterized by finger-like pores oriented perpendicular to the surface [60]. Examination of the surface micrograph reveals a homogeneous, smooth, and porous surface structure of the S substrate. For TFC membranes (after the formation of the PA layer on the substrate by the IP method), no such pores were found on their surfaces, indicating the uniformity of the formed and emerging PA layer. For these membranes, the formation of numerous low, evenly distributed nodular protrusions can be observed, which create a system of ridges and valleys. The similar surface “ridge-valley” structure has been reported previously in the works [41,79].

A PA layer with the thickness of about 840 nm was formed for the TFC SP membrane. The inclusion of the HKUST-1 modifier in the aqueous phase during the formation of the PA layer (the SPW5 membrane) resulted in a noticeable change in the morphology and thickness of the PA layer. As reported by J. Yin et al. [23], this behavior can be attributed to the altered rate of PA layer formation due to MOF presence. Thus, hydrophilic HKUST-1 forms hydrogen bonds with PIP, which leads to a reduction in the IP reaction rate. The introduction of HKUST-1 led to the development of the TFC membrane with a thinner PA layer (430 nm for SPW5 and 530 nm for SPO5) compared to the unmodified TFC SP membrane. This contributed to better permeability of the SPW5 membrane (Figure 2), and the SPW5 surface structure appears smoother compared to the SP membrane (confirmed by AFM data) [80]. However, an increase in productivity due to a decrease in the PA layer thickness compared to the SP membrane was not observed for the SPO5 membrane. This could be due to the introduction of MOF into the organic phase, causing the formation of a denser layer due to HKUST-1 agglomeration [64]. The PA layer for the SPO5 membrane was formed with separate high nodular protrusions on the membrane surface with the presence of voids and pinholes [66]. The formation of these defects and the increased layer thickness of the SPO5 membrane compared to the SPW5 membrane may be attributed to the following: the dissolution of polar MOF in a nonpolar solvent (naphtha) leads to HKUST-1 agglomeration, after which these agglomerates deposit on the substrate surface [64]. This results in PA layer formation within the substrate and creates void valleys in the active surface layer structure, as reported by R. Gayatri et al. [65]. This phenomenon is likely associated with agglomerate removal during the cooling process of the TFC membrane. Furthermore, study [81] also reported that fillers with relatively large sizes can significantly affect the integrity of the selective PA layer in TFC membranes. The SPWO5+5 membrane exhibits a similar surface morphology compared to the SPO5 membrane, but with a larger amount and size of protrusions. The increase in membrane PA thickness of the SPWO5+5 membrane compared to the SPW5 and SPO5 membranes may be due to the higher concentration of the micro-sized modifier (Figure S3 of the Supplementary Information). Increasing the concentration of the HKUST-1 modifier in the aqueous solution to 0.1 wt.% (SPW10) results in the formation of a thicker cross-sectional structure of PA layer and rougher surface with numerous high, narrow nodular protrusions of the PA layer compared to the SP and SPW5 membranes. Similar morphological changes in surface from “ridge and valley” to “ridge and valley-nodular” structures with increasing modifier concentration have been previously reported. This could cause decreased permeability and increased *R* and thickness (700 nm) due to the agglomeration of modifier particles [82].

Thus, although HKUST-1 agglomeration in SPO5 increased selectivity due to PA layer formation within the substrate, using the aqueous phase (SPW10) resulted in greater PA layer thickness compared to SPW5 because of agglomerate formation within the layer. In the second case, strategies that could help reduce agglomeration include the functionalization of MOFs [83], in situ interfacial synthesis of MOF [84], and optimization of incorporation methods [85].

**Figure 7:**
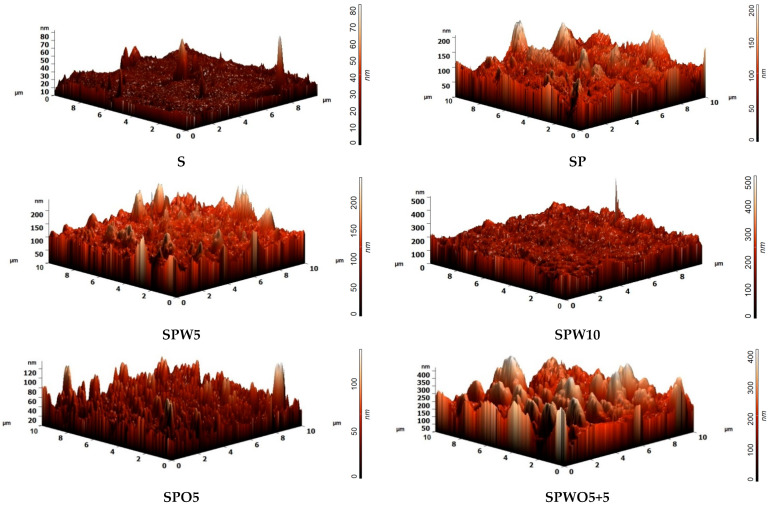
AFM images of surfaces of the S substrate and TFC membranes.

The surface topography was also investigated by AFM. Based on the AFM images presented in Figure 7, the average surface roughness (Ra) of the substrate and membranes was calculated. The Ra and contact angle values are summarized in Table 3.

When examining the surface topology, a significant increase in roughness for the TFC membranes compared to the S substrate was observed upon the formation of the PA layer with a “ridge-valley” structure (confirmed by SEM data). A similar effect was noted in studies [69,82,86]. In the case of the incorporation of 0.05% HKUST-1 (SPW5 and SPO5 membranes), the average roughness slightly decreased compared to the SP membrane. This change was associated with the densification of the PA layer and the formation of void valleys during modification with MOF (in agreement with SEM data, Figure 6). The introduction of the modifier in both the aqueous phase (WP) and organic phase (OP) (the SPWO5+5 membrane) resulted in significantly increased surface roughness compared to other membranes. This is due to the formation of individual high nodular protrusions (up to 740 nm) because of an increased MOF content in the formed PA layer and the formation of void valleys (confirmed by SEM data, Figure 6). Additionally, the surface of the SPW10 membrane was characterized by an increased Ra value compared to the SPW5 membrane due to a more developed “ridge and valley-nodular” structure (confirmed by SEM data, Figure 6).

The hydrophilic–hydrophobic balance of the surface of the S substrate and TFC membranes was evaluated by contact angle measurements. The deposition of the PA layer significantly enhanced the hydrophilicity (decreased water contact angle values) of the S substrate surface due to the formation of polar amide groups [53]. The introduction of HKUST-1 into the PA layer further reduced the contact angle for the TFC membranes due to its hydrophilic nature [42]. Furthermore, the utilization of HKUST-1 in the aqueous phase leads to a lower extent of TMC and PIP reaction, forming the PA layer [23], and an increased exposure of carboxylic acid groups on the surface from the hydrolysis of the acyl chloride group from TMC [87]. This may contribute to a decrease in the water contact angle of the SPW5 and SPW10 membranes compared to the SPO5 membrane. Y. Xu et al. [37] reported that the increase in MOF loadings led to a reduced extent of TMC and PIP cross-linking, confirming a greater hydrophilicity of SPW10 compared to SPW5. The SPWO5+5 membrane exhibits the lowest water contact angles, which may be attributed to the highest roughness and a presence of increased MOF content, as it is well known that the contact angle is closely related to surface roughness and material properties [88].

**Figure 8:**
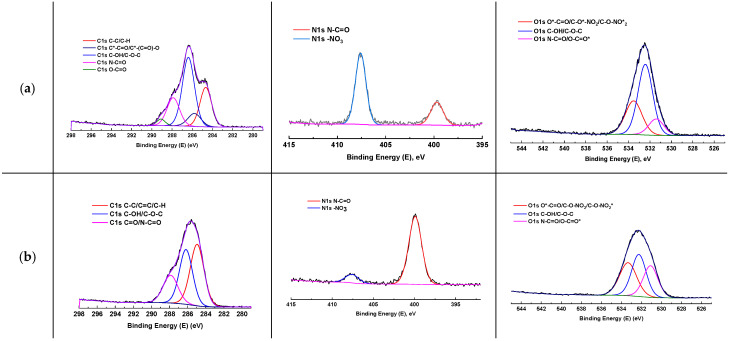
High-resolution XPS spectra of O1s, C1s, and N1s of the surface of the S substrate (**a**) and SPW5 (**b**) membranes. * indicates which atom of the functional group corresponds to the peak.

To gain a better understanding of the functional composition, the surface of the perspective TFC SPW5 membrane was studied by XPS. Figure 8 presents the high-resolution XPS spectra, and Table 4 displays the surface elemental compositions of the S substrate and SPW5 membranes for comparison.

Based on the data presented in Table 4, it was shown that the formation of the PA layer for SPW5 led to changes in the elemental composition ratios. Specifically, a decrease in oxygen content and an increase in carbon and nitrogen content were observed. This reduction was attributed to the low oxygen content in the PA layer compared to CN (C_6_H_7.75_O_9.5_N_2.25_) and CA (C_10_H_14_O_7_) and the high carbon content in the molecular frameworks of PIP (C_4_H_10_N_2_) and TMC (C_9_H_3_O_3_Cl_3_). Additionally, it was noted in work [37] that a decrease in the C/N and O/N ratios could indicate the formation of amide bonds and the corresponding PA layer.

The high-resolution XPS spectra (Figure 8) showed the C1s, N1s, and O1s orbital contributions of the membranes. The C1s spectrum of the CA/CN substrate displayed distinct peaks corresponding to cellulose ring carbons at 284.7 eV and methyl group carbons of cellulose acetate at 285.8 eV. An intensive peak observed at 286.4 eV was attributed to hydroxyl group carbons, ring carbons adjacent to oxygen atoms, and glycosidic bond carbons. Additional spectral features included N-C=O carbons from DMAc at 287.9 eV and acetate group carbons (O-C=O) at 289.1 eV [89]. In the O1s spectrum, the peak at 531.4 eV corresponds to amide oxygen (from solvent) and O-C=O* groups of acetate, while the peak at 532.5 eV is assigned to hydroxyl (OH) and ether (C-O-C) carbohydrate’s oxygens. The highest binding energy component at 533.6 eV originates from O*-C=O and nitrate group (-NO_3_) oxygens [89]. The deposition of the PA layer onto the S substrate resulted in a significant change in the signal profile, indicating alterations in the contributions of various components. In the case of the C1s spectrum, a notable enhancement of the signal corresponding to carbon species C-C, C=C, and C-H was observed, which aligned with the increase in carbon noted in the surface elemental composition (Table 4). For the S substrate, the XPS spectrum revealed signals corresponding to nitrogen from nitro groups and amide fragments (N-C=O) (residual solvent DMAc). The deposition of the PA layer onto the S substrate led to the substantial intensification of the amide signal, indicating the successful formation of the PA layer. At the same time, the residual signal from the nitro group suggested incomplete coverage of the S substrate surface, which was also noted in the work [20]. In the O1s spectra, the broadening of the signal (right shoulder of the peak) could be associated with the increasing contributions from amide and carboxylic fragments (from TMC molecules that did not react), which confirmed the conclusions regarding the decrease in hydrophilicity due to the formation of -COOH groups. Moreover, the general XPS survey (Figure S7 of Supplementary Materials) identified signals corresponding to copper ions (Cu^2+^). The increased copper content (0.13%) may indicate the migration of MOF particles to the surface of the PA layer, which is consistent with the results obtained from the contact angle measurements (Table 3).

### 3.3. Membrane Comparison and Perspectives

To assess the nanofiltration performance (in terms of water permeability and dye retention), Table 5 presents a summary comparing established commercial membranes or membranes described in the literature with the TFC membranes (SPW5 and SPO5) developed in this study.

It is shown that the developed TFC membranes have higher permeability and lower dye rejection compared to most membranes described in the literature. The data presented in Table 5 confirm that, for most of membranes, there is a selectivity (rejection)-permeability trade-off [100]. However, the developed membranes are promising for the industrial nanofiltration process of water purification from dyes; namely, the high permeability of the SPW5 membrane will increase the productivity of the process, and the high selectivity of the SPO5 membrane with a sufficient level of permeability will increase the selectivity of the process. Moreover, the mTFC membranes have demonstrated exceptional heavy metal ion rejection, further expanding their applicability in water treatment.

Despite these advantages, scaling up TFC membrane production from laboratory to industrial levels presents considerable challenges, particularly in monomer synthesis, its application, and IP process control [101]. A critical limitation lies in the insufficient understanding of monomer diffusion–reaction kinetics during IP, which can result in mismatches between heating durations and polymerization rates, ultimately affecting membrane uniformity. Additionally, large-scale monomer production involves substantial costs due to complex purification procedures and strict quality control requirements. To overcome these barriers, future research should prioritize a deeper investigation of monomer transport-reaction mechanisms during IP, improved characterization techniques for TFC membranes, and the development of more economical monomer purification protocols [101].

## 4. Conclusions

Nanofiltration TFC membranes obtained by forming a selective PA layer on the surface of a developed CA/CN blend substrate via the IP technique for enhanced water treatment of dyes were developed. The successful formation of a uniform PA layer on the substrate was confirmed by FTIR and XPS spectroscopies.

Efficient TFC membranes were subsequently produced by incorporating HKUST-1 into the PA layer, introducing various HKUST-1 concentrations (0.05 and 0.1 wt.%) into the aqueous and/or organic phase during the formation process. It was demonstrated that adding 0.05 wt.% HKUST-1 to the aqueous phase (SPW5) significantly enhanced permeability, reaching 123 LMH bar^−1^, while its incorporation into the organic phase (SPO5) achieved the highest dye rejection, exceeding 89%. These changes were attributed to significant alterations in the morphology and surface properties of the PA layer due to the incorporation of the porous HKUST-1 into the aqueous or organic phase, as evidenced by SEM, AFM, and contact angle data. The application of this modifier provided additional transport channels for water molecules due to its porous structure, along with changes in morphology, surface roughness, layer thickness, and improved hydrophilicity of the membranes. The use of HKUST-1 in the organic phase led to its agglomeration, which appeared to create a distinct surface layer topology. This, in turn, hindered mass transport through the PA layer and increased retention. Increasing the HKUST-1 content to 0.1 wt.% (either in the aqueous phase at 0.1 wt.% or in both phases at 0.05 wt.%) led to particle agglomeration, which resulted in a decrease in permeability and a modest rise in rejection coefficients. Additionally, the optimal membranes SPW5 and SPO5 demonstrated effective retention of heavy metal ions (Cu^2+^, Cd^2+^, Pb^2+^) exceeding 91.7% compared to the pristine SP membrane.

Thus, the developed SPW5 and SPO5 TFC membranes were identified as optimal and promising for the industrial nanofiltration water treatment of dyes. It was found that, by adjusting the method of incorporating HKUST-1 into the PA layer, the developed mTFC membranes could be optimized to either improve productivity or enhance selectivity in the water purification process. However, several questions with respect to their potential upscaling remain to be addressed through further research, including the assessment of MOF large-scale production [102] and dispersion stability [103], and the following main criteria for the industrial valorization of novel membranes [104]: industrial operating conditions such as resistance to chlorinating agents and the retention capacity for other organic pollutants and salts, long-term membrane stability, capital and operating expenditure analysis, and waste management.

## Figures and Tables

**Table 1:** The structures, absorption maxima, and molar masses of the dyes.

Dyes	Molecular Formula	Structure	Absorption Maximum, nm	Molar Mass, g mol^−1^
SY	C_16_H_10_N_2_Na_2_O_7_S_2_	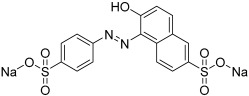	483	429
CR	C_32_H_22_N_6_Na_2_O_6_S_2_	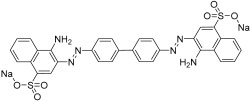	505	697
AZ	C_37_H_34_Na_2_N_2_O_9_S_3_	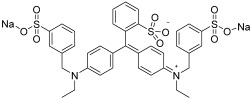	628	793

**Table 2:** Abbreviation and composition of membranes.

Membrane	CA/CN-20 (1:3) Substrate	PA Layer	HKUST-1 Phase	HKUST-1 Concentration, wt.%
S	+	−	−	0
SP	+	+	−	0
SPO5	+	+	Organic	0.05
SPW5	+	+	Water	0.05
SPW10	+	+	Water	0.1
SPWO5+5	+	+	Organic + water	0.05 + 0.05

**Table 3:** Average surface roughness and contact angle.

Membrane	Ra, nm	Contact Angle, °
S	1.9	45 ± 2
SP	16.9	26 ± 2
SPW5	14.9	19 ± 2
SPO5	9.4	25 ± 2
SPWO5+5	50.7	17 ± 2
SPW10	21.0	18 ± 2

**Table 4:** Surface elemental composition.

Membrane	Cu, %	C, %	O, %	N, %	C/N	O/N
S	0	56.51	36.11	7.38	7.66	4.89
SPW5	0.13	64.54	24.99	10.34	6.24	2.42

**Table 5:** A performance comparison in nanofiltration of anionic dye solutions.

NF Membranes *	*L*, LMH bar^−1^	Dye *	MM, g mol^−1^	*R*, %	Reference
SPW5	123	SY	429	76	This study
SPO5	28	SY	429	89	This study
TFC-(6FAPBS/6FBABDS+PIP+TMC)/PPSU	9	Methyl Orange	327	76	[90]
TFC-PEI-g-SBMA	13.2	Orange GII	452	90	[91]
TFC-PPSU/PEG-PPG-PEG	4.8	Indigo carmine	466	90	[20]
Sulfated CMC	6.6	Xylenol orange	673	90	[92]
SPW5	123	CR	697	78	This study
SPO5	28	CR	697	96	This study
TFC-(sericin-TMC)	11.9	CR	697	>99	[93]
TFC-PSSNa/PVA–PSF	8.3	CR	697	>99	[94]
Sepro NF 2A	10.5	CR	697	>99	[95]
mTFC-mZIF	14.9	RB2	774	99	[69]
SPW5	123	AZ	793	75	This study
SPO5	28	AZ	793	90	This study
SPECMs	6.7	Methyl blue	800	>99	[96]
mTFC-mZIF	14.9	RB5	992	99	[69]
TFC-SR2	11.8	RB5	992	99	[97]
QPEI-PES	12.6	RB5	992	97	[98]
HNTs-PIL/PES	11.6	RB5	992	95	[99]

* Modified ZIF-8 (mZIF); carboxymethyl cellulose (CMC); sulfated polyelectrolyte complex membranes (SPECM); poly(ether sulfone) (PES); quaternized polyethylenimine (QPEI); halloysite nanotubes (HNTs); poly (ionic liquid) (PIL); poly(vinyl alcohol) (PVA); poly(sodium-p-styrene-sulfonate) (PSSNa); sulfobetaine methacrylate (SBMA); polyethylenimine (PEI); 2,5-bis(4-amino-2-trifluoromethyl-phenoxy)benzenesulfonic acid (6FAPBS); 4,4′-bis(4-amino-2-trifluoromethyl-phenoxy)biphenyl-4,4′-disulfonic acid (6FBABDS); polyphenylene sulfone (PPSU); polyvinylpyrrolidone (PVP); Reactive blue 2 (RB2); Reactive Black 5 (RB5).

## Data Availability

Data are contained within the article or Supplementary Materials.

## Supplementary Materials

The following supporting information can be downloaded at: https://www.mdpi.com/article/10.3390/polym17091137/s1, S1: Materials; S2: Preparation of HKUST-1; S3: HKUST-1 Investigation; S4: Dye molecule size; S5: Membrane Investigation; Figure S1: XRPD of HKUST-1, alongside the simulated XRPD pattern based on the cif data.; Figure S2: Low-temperature N2 adsorption isotherm of HKUST-1.; Figure S3: SEM image of the HKUST-1.; Figure S4: FTIR spectrum of the HKUST-1.; Figure S5: The size of dye molecules based on the van der Waals radii of atoms.; Figure S6: Permeation flux as a function of transmembrane pressure.; Figure S7: XPS spectrum of SPW5 membrane. References [46,105,106] are cited in the Supplementary Materials.
