# Supplementary material for: Thin-Film Composite Polyamide Membranes Modified with HKUST-1 for Water Treatment: Characterization and Nanofiltration Performance
Source: Polymers (Basel). 2025 Apr 22;17(9):1137. doi: 10.3390/polym17091137 (PMC12073345; doi:10.3390/polym17091137)
Supplement: Supplementary file 1 [file polymers-17-01137-s001.zip › polymers-3551289-supplementary.pdf]

Thin-film Composite Polyamide Membranes Modified with HKUST-1 for Water Treatment: Characterization and Nanofiltration performance

Roman Dubovenko [1,*], Mariia Dmitrenko [1,*], Anna Mikulan [1], Margarita Puzikova [1], Ilnur Dzhakashov [1], Nadezhda Rakovskaya [1], Anna Kuzminova [1], Olga Mikhailovskaya [1], Rongxin Su [2] and Anastasia Penkova [1,*]

1 St. Petersburg State University, 7/9 Universitetskaya nab., St. Petersburg 199034, Russia;
2 State Key Laboratory of Chemical Engineering, School of Chemical Engineering and Technology, Tianjin University, Tianjin 300072, China; surx@tju.edu.cn (R.S.)
* Correspondence: r.dubovenko@spbu.ru (R.D.); m.dmitrienko@spbu.ru (M.D.); a.penkova@spbu.ru (A.P.); Tel.: +7-(812)-363-60-00 (ext. 3367) (R.D., M.D. & A.P.)

S1. Materials

The following materials were used without further purification: benzene-1,3,5-tricarboxylic acid (Trimesic acid, BTC) from Sigma-Aldrich, St. Louis, MO, USA, cupric nitrate trihydrate and ethanol from LenReactiv and Vecton, St. Petersburg, Russia.

S2. Preparation of HKUST-1

HKUST-1 was synthesized similarly to the procedure presented in the work [105]: 2.49 g of cupric nitrate trihydrate was dissolved into 100 mL of deionized water. Then 100 mL of an aqueous-alcoholic solution (water:ethanol (1:1)) of BTC (1.6 g) was added to the resulting mixture. The resulting solution was transferred to a Teflon-lined autoclave and heated to 110 °C for 18 h. Then the precipitated solid was filtered, washed three times with a mixture of deionized water and ethanol and dried in vacuum at 110 °C.

S3. HKUST-1 Investigation

The X-ray Diffraction

The crystal structure of the synthesized HKUST-1 was characterized using the X-ray powder diffraction (XRD) technique. To acquire the XRD patterns, a Bruker "D8 DISCOVER" high-resolution diffractometer utilizing Cu Kα radiation was employed, covering a 2θ range of 5 to 60°. The resulting XRD pattern is illustrated in Figure S1.

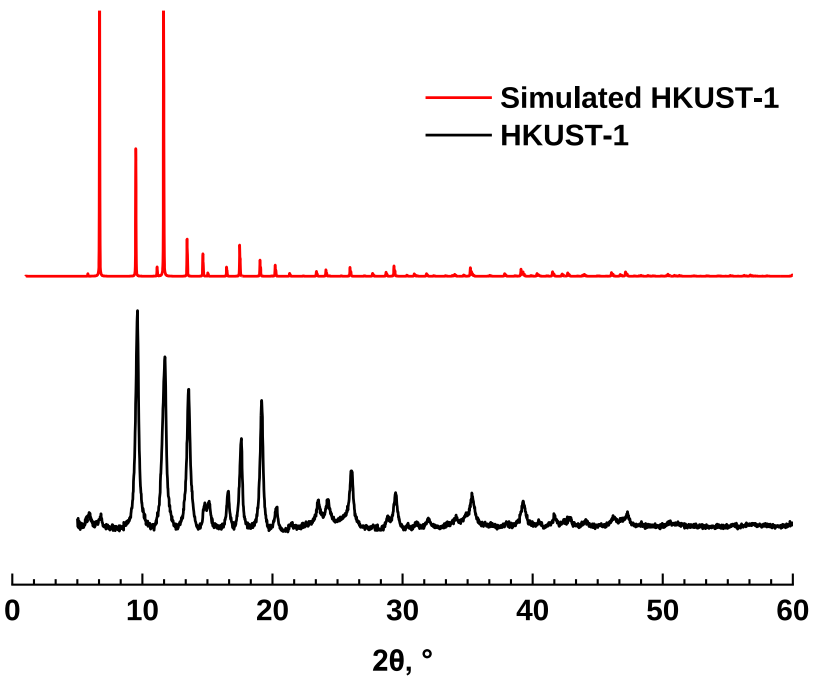

Figure S1. XRD pattern of HKUST-1 and the simulated XRD pattern based on the cif data.

The XRD pattern obtained for the synthesized HKUST-1 shows a strong correlation with the XRD pattern computed from its crystal structure [46], confirming the formation of the HKUST-1 phase.

Low-temperature Nitrogen Adsorption

The specific surface area of the synthesized HKUST-1 were assessed through low-temperature nitrogen adsorption analysis. This analysis was carried out using an automated ASAP 2020MP system (Micromeritics, USA). The nitrogen adsorption isotherm for HKUST-1 is presented in Figure S2.

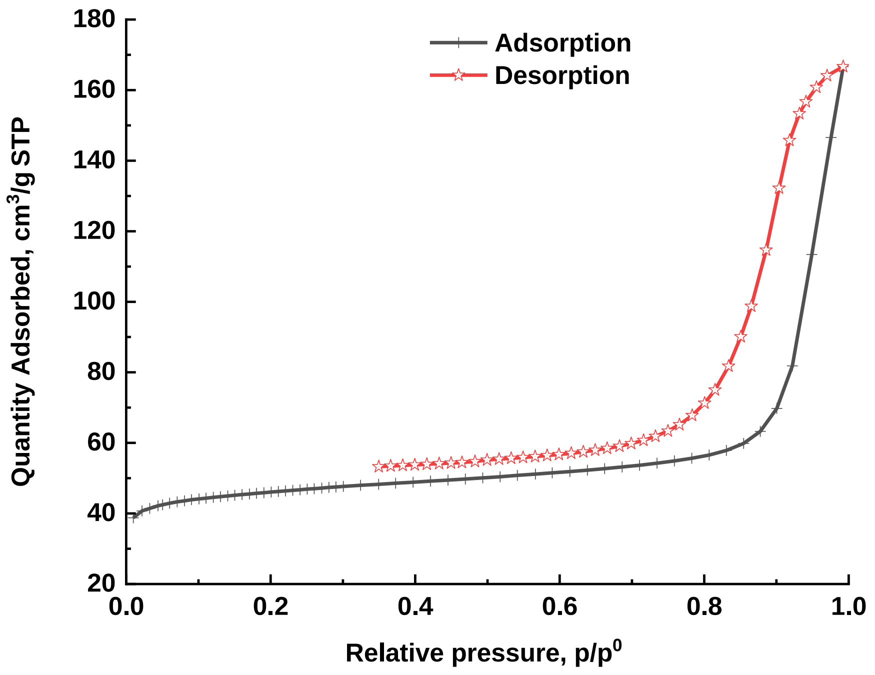

Figure S1. Low-temperature N_2 adsorption isotherm of HKUST-1.

The specific surface area was equal to 171.6±0.9 m^2 g^{-1} for HKUST-1.

Scanning Electron Microscopy

The morphology of the synthesized HKUST-1 was examined using a Zeiss AURIGA laser scanning electron microscope (Carl Zeiss SMT, Oberkochen, Germany). The resulting SEM images are displayed in Figure S3.

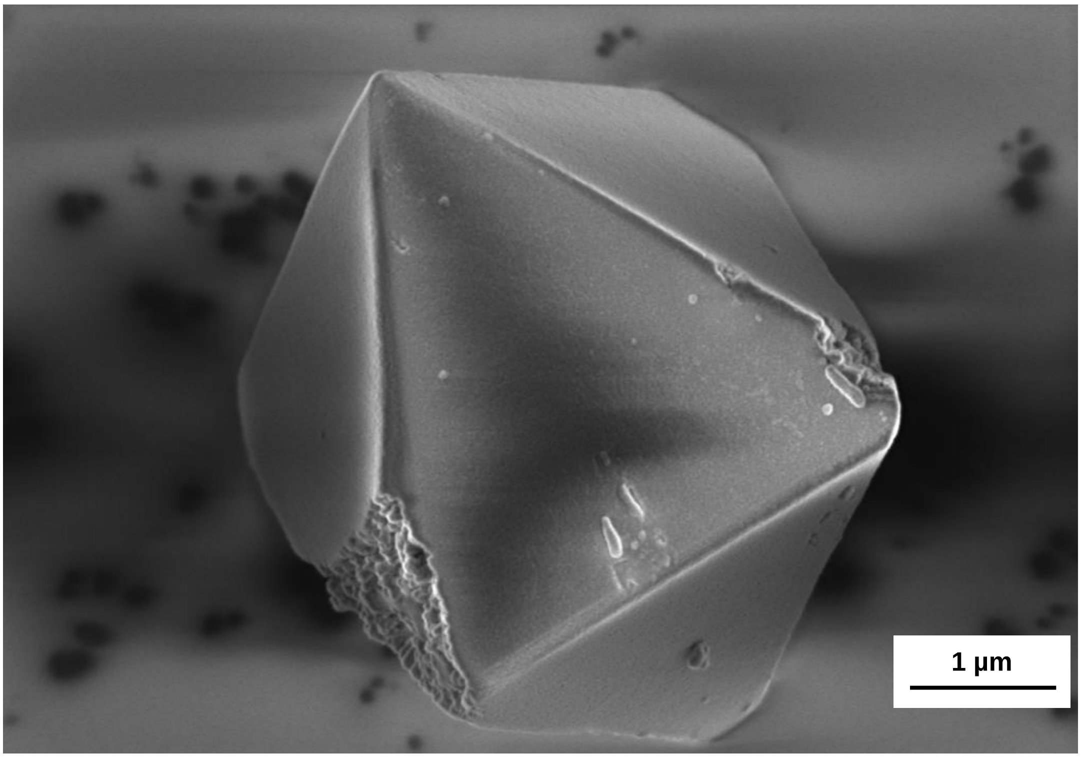

Figure S2. SEM image of the HKUST-1.

Based on SEM images, it was shown that HKUST-1 has an octahedral shape. The average diameter is approximately 4 µm.

Fourier Transform Infrared Spectroscopy

The FTIR data was collected by the IRAffinity-1S spectrometer (Shimadzu, Kyoto, Japan). KBr pellets in transmission mode were used for powder samples, and attenuated total reflection (ATR) mode was used. The FTIR spectrum of HKUST-1 is illustrated in Figure S4.

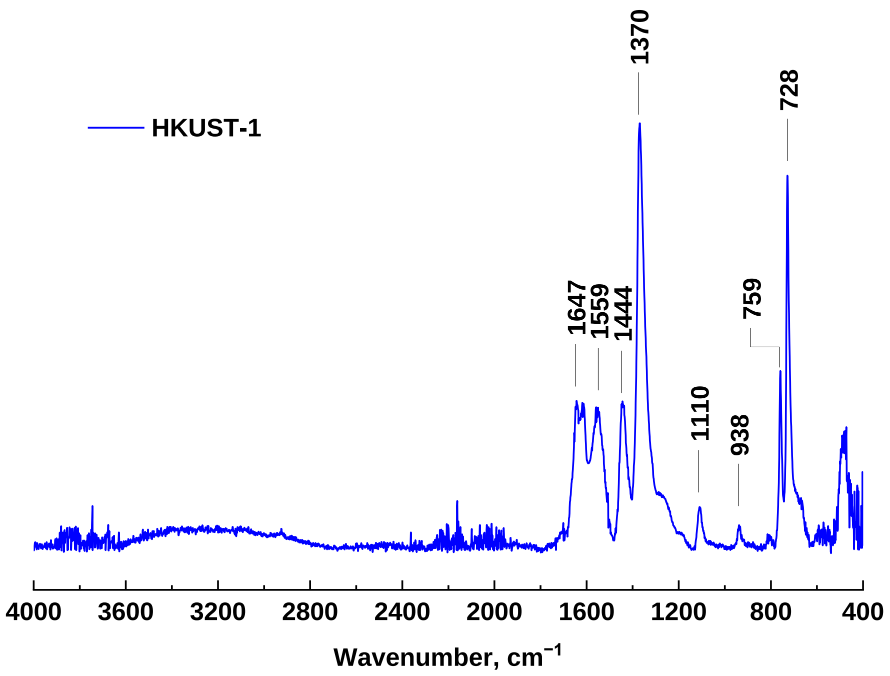

Figure S4. FTIR spectrum of the HKUST-1.

The bands ranging from 1350 to 1700 cm^{-1} were associated with two distinct types of carboxylate groups, which corresponded to the coordination of BTC with the copper sites. Peaks near 1647 cm^{-1} was linked to the asymmetric stretching vibrations of the carboxylate in BTC, while the peaks at 1444 and 1370 cm^{-1} were associated with the symmetric stretching vibrations of the carboxylate groups. Overall, the obtained spectrum aligns with the spectrum previously described in the work [106].

S4. Dye Molecule Size

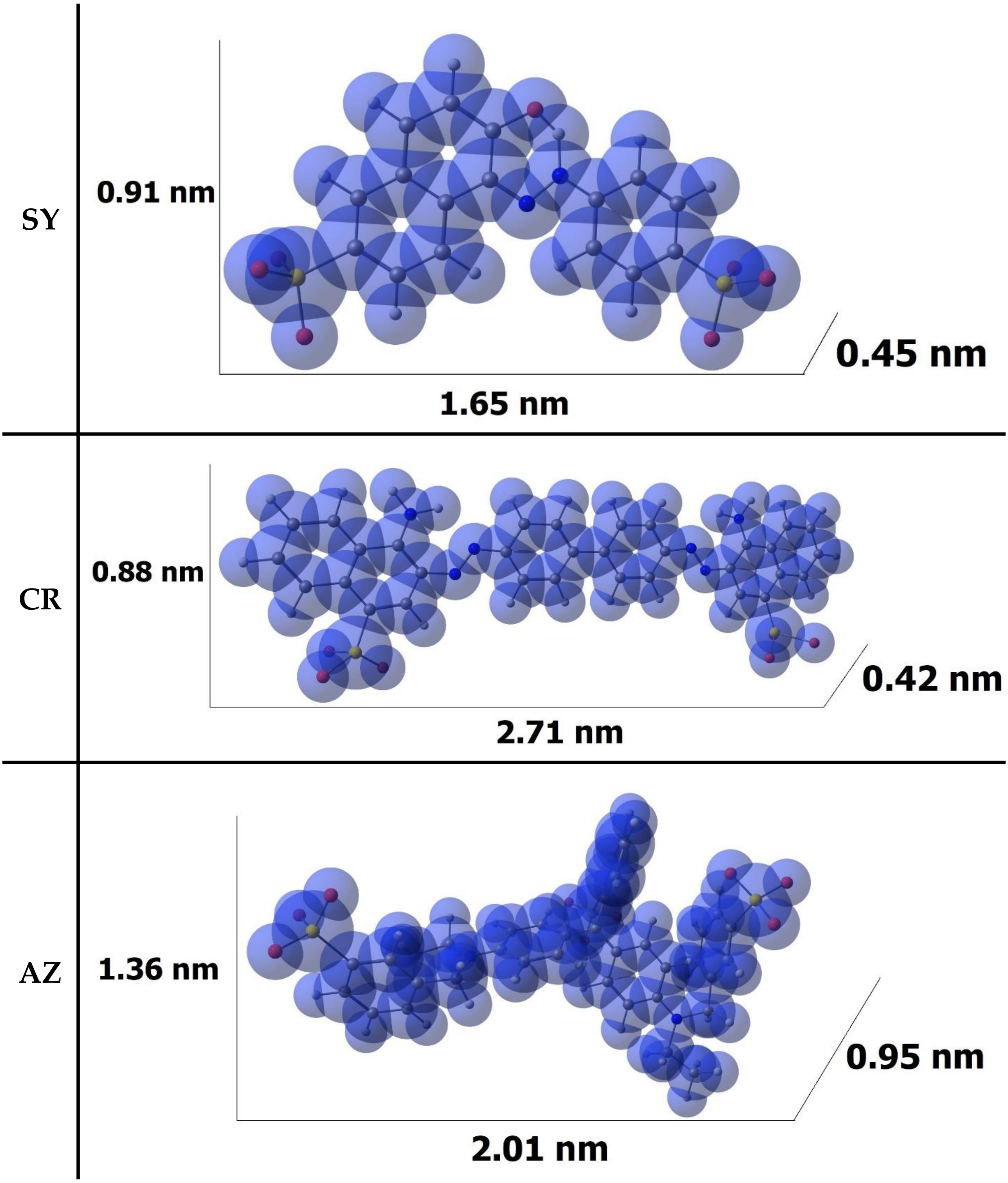

Figure S5. The size of dye molecules based on the van der Waals radii of atoms.

S5. Membrane Investigation

The obtained SP, SPW5, and SPO5 membranes were investigated to examine the dependence of permeation flux on transmembrane pressure (Figure S6).

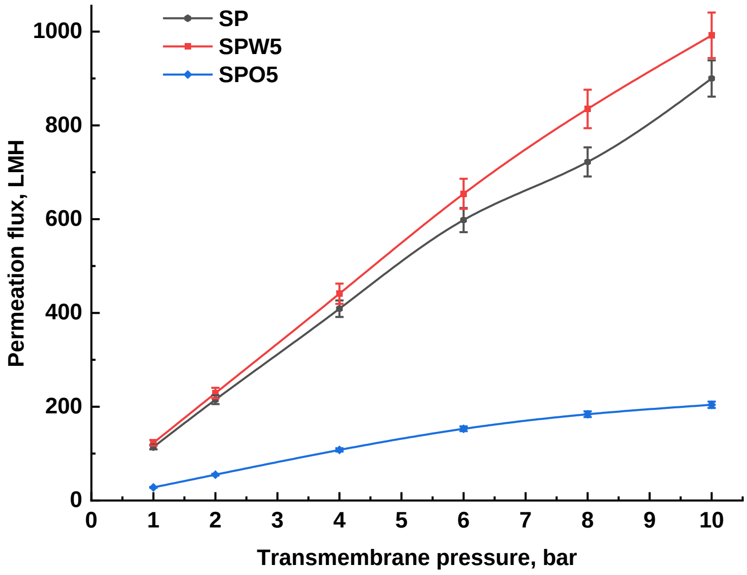

Figure S6. Permeation flux as a function of transmembrane pressure.

The XPS spectrum is presented in Figure S7, which also indicates the presence of the HKUST-1 modifier on the surface of the SPW5 membrane and is consistent with the observed effect of surface hydrophilization (confirmed by contact angle measurements).

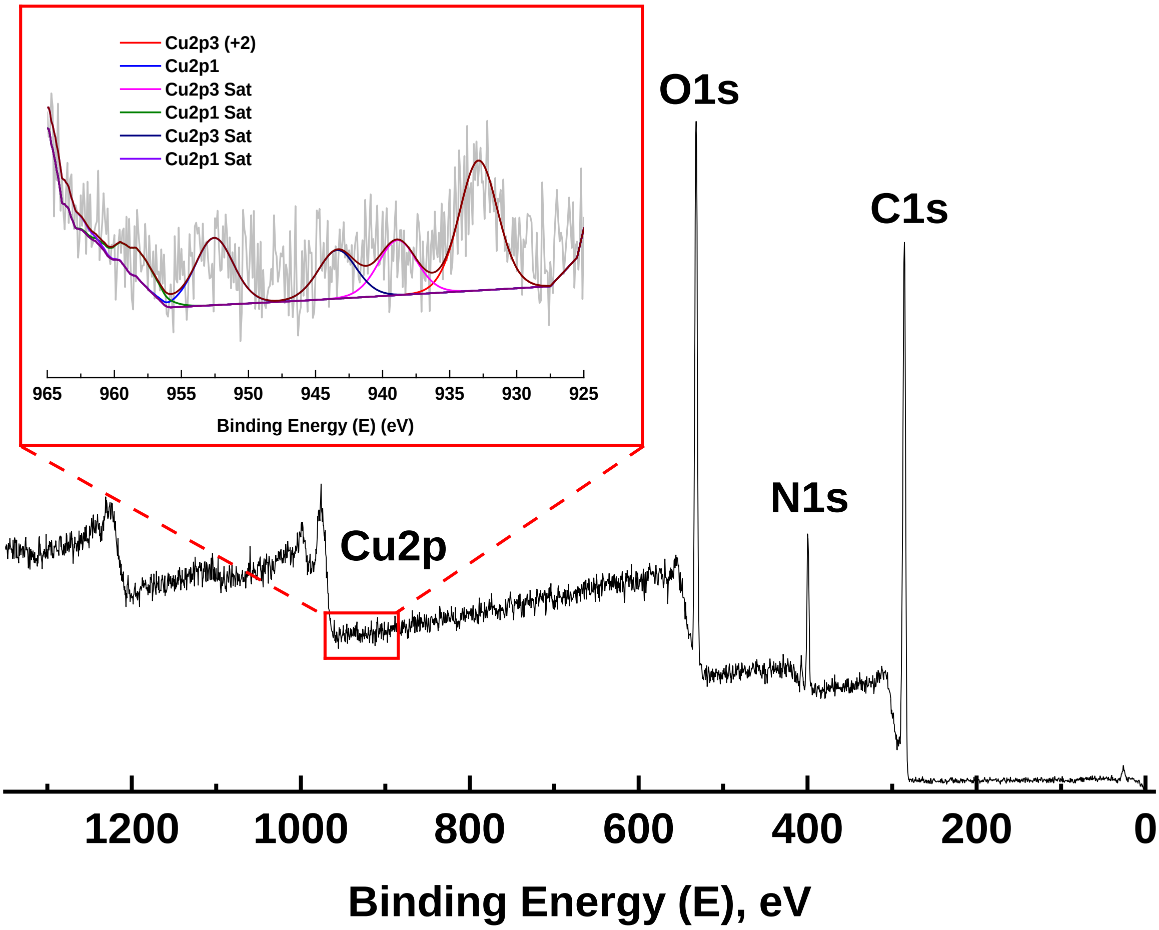

Figure S7. XPS spectrum of the SPW5 membrane.

References

[46] S.S.-Y. Chui, S.M.-F. Lo, J.P.H. Charmant, A.G. Orpen, I.D. Williams, A Chemically Functionalizable Nanoporous Material $[Cu_3(TMA)_2(H_2O)_3]_n$, Science (1979) 283 (1999) 1148–1150. https://doi.org/10.1126/science.283.5405.1148.

[105] K.-S. Lin, A.K. Adhikari, C.-N. Ku, C.-L. Chiang, H. Kuo, Synthesis and characterization of porous HKUST-1 metal organic frameworks for hydrogen storage, Int J Hydrogen Energy 37 (2012) 13865–13871. https://doi.org/10.1016/j.ijhydene.2012.04.105.

[106] F.S. Gentile, M. Pannico, M. Causà, G. Mensitieri, G. Di Palma, G. Scherillo, P. Musto, Metal defects in HKUST-1 MOF revealed by vibrational spectroscopy: a combined quantum mechanical and experimental study, J Mater Chem A Mater 8 (2020) 10796–10812. https://doi.org/10.1039/D0TA01760D.
